# *QuickStats:* Age-Adjusted Death Rates* for Lung Cancer,^†^ by Urbanization of County of Residence^§^ — National Vital Statistics System, United States, 2006 and 2016

**DOI:** 10.15585/mmwr.mm6736a8

**Published:** 2018-09-14

**Figure:**
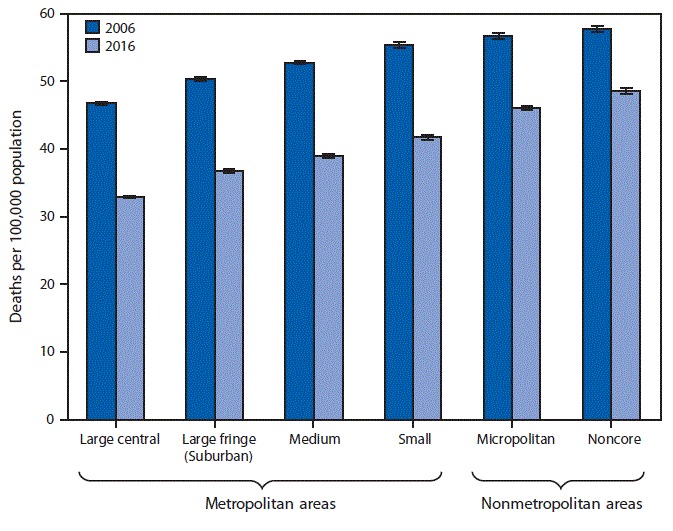
From 2006 to 2016, the age-adjusted death rate for lung cancer decreased in each of the six urbanization levels, with the largest decrease (29%) in large central metropolitan counties and the smallest decrease (16%) in noncore counties. In both years, the rate of lung cancer death was higher in nonmetropolitan areas than in metropolitan areas. In 2016, the lung cancer death rate in noncore counties was 48.6 per 100,000 compared with 33.0 in large central metropolitan counties.

**Sources:** National Center for Health Statistics, National Vital Statistics System, Mortality data. https://www.cdc.gov/nchs/nvss/deaths.htm; 2013 National Center for Health Statistics urban-rural classification scheme for counties. https://www.cdc.gov/nchs/data/series/sr_02/sr02_166.pdf.

